# Acidic Potassium Dichromate Solutions as Ultraviolet Absorbance Standards*

**DOI:** 10.6028/jres.080A.062

**Published:** 1976-08-01

**Authors:** R. W. Burke, R. Mavrodineanu

**Affiliations:** Institute for Materials Research, National Bureau of Standards, Washington, D.C. 20234

**Keywords:** Absorbance linearity, accuracy, acidic potassium dichromate solutions, calibration of ultraviolet spectrophotometers, liquid filters, transfer standards, ultraviolet absorbance standards

## Abstract

The absorbances of five concentrations of potassium dichromate in 0.001 *M* perchloric acid have been determined at eight wavelengths in the ultraviolet on the National Bureau of Standards Institute for Materials Research high-accuracy spectrophotometer. Four of the wavelengths—235, 257, 313, and 350 nm—correspond to absorbance maxima or minima in the HCrO_4_^–^ spectrum and are useful wavelengths for checking the accuracy of the absorbance scale of narrow bandpass spectrophotometers. Although partial dimerization of HCrO_4_^–^ to Cr_2_O_7_^=^ produces small positive deviations from Beer’s law at these wavelengths, the apparent absorptivities calculated for each concentration are reproducible to one part in a thousand. The estimated uncertainties in the absorptivity values are ± 0.7 percent at 0.1 absorbance (*A*) and ± 0.2 percent near *A* = 1. These uncertainties include all known sources of possible systematic error and the 95 percent confidence level for the mean. The remaining four wavelengths used for measurement are near two predicted isosbestic points in the HCr0_4_^–^/Cr_2_O_7_^=^ spectra. The absorptivities at 345 nm are sufficiently independent of concentration that this wavelength can be used for checking absorbance linearity to one part in a thousand over the range *A* = 0.2–1.

## I. Introduction

At present, there are no certified standards available from the National Bureau of Standards (NBS) for checking the accuracy of the absorbance scale of spectrophotometers throughout the ultraviolet. The number of analytical applications in this important region of the spectrum, however, continues to increase relatively sharply and the need for such standards is becoming increasingly acute.

The area with perhaps the most serious need for ultraviolet absorbance standards at the moment is clinical chemistry. Workers in this field, for example, frequently use molar absorptivity as an index of purity of their biological standards. Only recently, Burnett [1]^1^ has discussed the importance of accurate molar absorptivity measurements in the clinical laboratory. He especially emphasizes the need for spectrophotometric accuracy in clinical enzymology. Not only must the molar absorptivity of the substrate or enzyme-catalyzed reaction product be known accurately but also the individual absorbance measurements on the test sample must be accurate. This dual requirement for accuracy in this instance arises because high-purity and well- characterized enzyme preparations are not yet routinely available for use as standards.

A program has been under way at NBS in the Institute for Materials Research (IMR) since 1969 for the development and issuance of visible and ultraviolet transfer standards for use as Standard Reference Materials (SRM’s). Two such standards are currently available: (1) SRM 930, consisting of a set of three neutral Schott NG glass filters and (2) SRM 931, an empirical inorganic solution available at three concentrations in 10-ml ampoules. Neither of these SRM’s meets the present needs for absorbance standards in the ultraviolet. The glass filters do not transmit below about 350 nm while the inorganic solution is certified only at one wavelength in the ultraviolet (302 nm) and with an uncertainty of ± 1 percent. Transfer standards capable of being certified throughout the range of 200–350 nm and with a smaller uncertainty are being investigated, however. The most promising solid filter at the moment for this purpose is the metal-on-quartz type. Some recent experiences with this filter are discussed in the paper immediately following [2]. Of the chemical or liquid-type filters that have been proposed, the weakly acidic potassium dichromate system is considered the best. A critical evaluation of this system, as well as several other candidate materials, was presented in a previous paper [3].

In this paper, we present absorptivity values for five concentrations of potassium dichromate in 0.001 *M* perchloric acid. These concentrations effectively span the absorbance range of *A* = 0.1–1.5 when measured in 10 mm cuvettes. The absorptivities were calculated from transmittance measurements at eight wavelengths in the ultraviolet on the IMR high-accuracy spectrophotometer. Four of the wavelengths—235, 257, 313 and 350 nm—correspond to absorbance maxima and minima in the HCrO_4_^–^ spectrum and are useful wavelengths for checking the accuracy of the absorbance scale of narrow bandpass spectrophotometers. Although partial dimerization of HCrO_4_^–^ to Cr_2_O_7_^=^ produces small positive deviations from Beer’s law at these wavelengths, the apparent absorptivities calculated for each concentration are reproducible to one part in a thousand. The estimated uncertainties in the absorptivity values are ± 0.7 percent at 0.1 absorbance (*A*) and ± 0.2 percent near *A* = 1. These uncertainties include all known sources of possible systematic error and the 95 percent confidence level for the mean. The remaining four wavelengths used for measurement are near two predicted isosbestic points in the HCrO_4_^–^/Cr_2_O_7_^=^ spectra. The absorptivities at 345 nm are sufficiently independent of concentration that this wavelength can be used for checking absorbance linearity to one part in a thousand over the range *A* = 0.2–1.

## II. Experimental Procedure

The potassium dichromate (K_2_Cr_2_O_7_) used in this study was a special lot of analytical reagent grade material obtained from the J. T. Baker Chemical Company.^2^ Drying studies performed at 105 °C indicated that the surface moisture of this material was less than 0.01 percent. No measurement of possible occluded water was made. However, a recent paper [4] by Yoshimori and Sakaguchi has shown that K_2_Cr_2_O_7_ typically contains 0.01 to 0.02 percent occluded water which can be removed only by crushing and drying at 350 °C.

Coulometric assay of the undried potassium dichromate gave a purity, expressed as an oxidant, of 99.972 ± 0.005 percent at the 95 percent confidence level. Emission spectroscopy indicated that the only significant elemental impurities present were sodium and rubidium. Their concentrations were estimated to be 0.02 and 0.03 percent, respectively.

The concentrations of the K_2_Cr_2_O_7_ solutions used throughout this study are expressed on a weight/weight basis. Milligram samples of K_2_Cr_2_O_7_ were weighed to the nearest microgram on a microbalance. After dissolution in distilled water shown to be free of reducing impurities (see Discussion), 1 ml of 1 *M* perchloric acid was added and the solutions were diluted approximately to volume in 1-liter volumetric flasks. Each flask was fitted with a doubleribbed Teflon stopper (Kontes Glass Company, Vineland, New Jersey) to prevent evaporation. The weight of each solution was determined on a singlepan top-loading balance having a sensitivity of 0.01 g. NBS-calibrated weights were used to establish the accuracy of the balances. The concentrations of the solutions were then calculated after correcting all weights to vacuum. A solvent blank was prepared by diluting 1 ml of 1 *M* perchloric acid to 1 liter with distilled water.

The Institute for Materials Research high-accuracy spectrophotometer was used for performing the transmittance measurements which, in turn, were converted to absorbance. The design and construction of this instrument have been described in detail by one of us (RM) in reference [5] and will not be repeated here. Similarly, the quartz cuvettes used are also of NBS design and construction and have been described previously [6]. These cuvettes are currently available through the NBS Office of Standard Reference Materials as SRM 932. Each cuvette is certified for path length and parallelism to ± 0.0005 mm.

Prior to use the cuvettes were cleaned by soaking in concentrated (18 *M*) sulfuric acid for several hours. In order to minimize the heat of mixing, they were then transferred consecutively to 12, 6 and 3 *M* sulfuric acid before rinsing with distilled water. After rinsing thoroughly, the cuvettes were air-dried under an inverted Petri dish that served as a dust-protective cover.

For sample measurements, five calibrated cuvettes were placed in separate holders in the rotating sample compartment [5] of the IMR high-accuracy spectrophotometer and a reference filter (inconel-on-quartz) was placed in a sixth position. The cuvettes were left in their respective holders for the duration of the experiment. All transmittance/absorbance measurements were made relative to air in a temperature-controlled room at 23.5 ± 0.3 °C. The transfer of solvent blank and sample solutions to and from these cuvettes was made by means of borosilicate, Pasteur-type, disposable pipettes. After being rinsed with the test solution, a final transfer for measurement could be made in 10–15 seconds, after which time the cuvette was immediately stoppered with a snugly-fitting Teflon stopper.

Six sets of solutions having nominal concentrations of 20, 40, 60, 80 and 100 mg K_2_Cr_2_O_7_/kg were prepared. Each concentration within a set was measured a minimum of six times at the eight wavelengths of interest. The absorbance, *A* = −log (*T*_Sample_/*T*_Blank_), was computed for each wavelength and concentration from the average of the six transmittance measurements (*T*). Absorptivities were then calculated after correcting the absorbances for systematic errors due to cuvette path length, beam geometry and internal multiple reflections. No correction was applied for the reflections discussed by Mielenz and Mavrodineanu [7] from internal components such as the lenses and slit jaws because these are adequately compensated for by the blank. A detailed account of these corrections and calculations will be given in an NBS 260 Special Publication which is now in preparation.

## III. Discussion and Results

**Figure 1:**
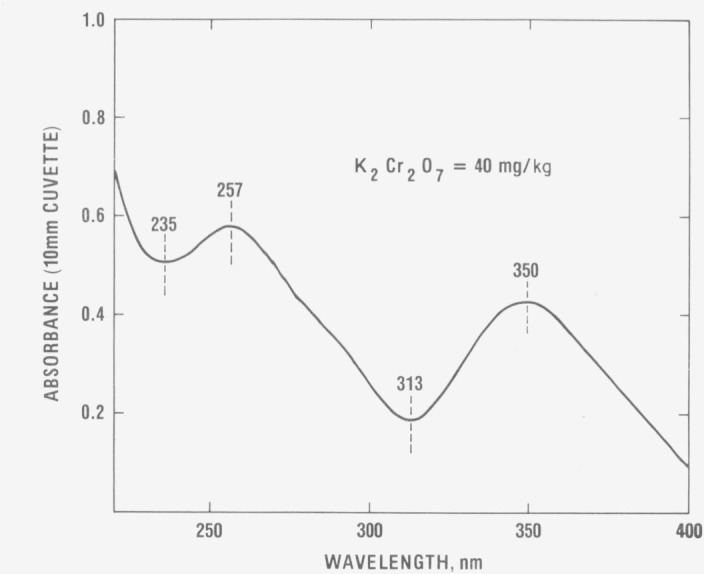
Absorbance spectrum of *K_2_Cr_2_O_7_* in 0.001 *M* perchloric acid.

The ultraviolet absorbance spectrum of a 40 mg kg^−1^ solution of potassium dichromate in 0.001 *M* perchloric acid is shown in figure 1. The four wavelengths selected for certification of absorptivity of this absorbing system, namely 235, 257, 313 and 350 nm, are also indicated. The maxima and minima are sufficiently broad that serious restrictions are not placed upon instrumental spectral bandwidth requirements. The half bandwidths of the 257 and 350 nm peaks, for example, are approximately 60 nm so that an instrumental spectral bandwidth of 3 nm or less is sufficient for obtaining at least 0.999 of the maximum peak intensities.

**Figure 2:**
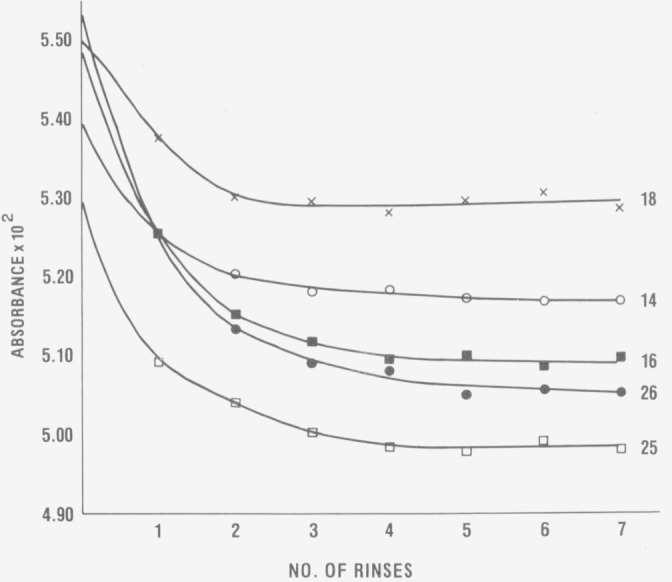
Apparent absorbances of the blank solvent in the five curvettes used—Nos. 14, 16, 18, 25, and 26 ref. [6].

The first experiment performed on the IMR high- accuracy spectrophotometer was the determination of the rinse behavior and reproducibility of the transmittance/absorbance measurements of the solvent blank. In the initial studies, measurements were made at 235 nm only, since any problems associated with the transfer of solution were expected to be the greatest at the shortest wavelength used. The results obtained for the five cuvettes employed are shown in figure 2. All exhibit a similar rinse pattern, attaining a minimum and constant absorbance value after 5–6 rinses (the term rinse as used here and throughout the remainder of this paper refers to the exchange of one cuvette volume for a second one). Once the blank measurements were in control, similar absorbance measurements were then made on the first series of K_2_Cr_2_O_7_ solutions. Five concentrations having nominal concentrations of 20, 40, 60, 80 and 100 mg K_2_Cr_2_O_7_/kg were measured. Table I summarizes the results of these measurements together with the final solvent blank values for 235 nm. The cycle of measurements shown here was then extended to the other seven wave-lengths of interest. In all, six sets of K_2_Cr_2_O_7_ solutions were measured. Typically, two volleys consisting of three transmittance/absorbance measurements were made on each of the 30 solutions. The first volley was made after rinsing the cuvettes, initially containing solvent, with five rinses of sample and was repeated again after two additional rinses. Since no systematic increase in absorbance was ever observed when the results of the first volley of measurements were compared to the second, the six measurements were invariably averaged. The absorbances were obtained by subtracting the solvent blank values from the sample readings. After applying appropriate corrections for beam geometry and internal multiple reflections, the corrected absorbances (*A*_corr_) were used to calculate the desired absorptivities, using the relationship

$$\text{Absorptivity}=A_{\text{corr}}/bc,$$

where *b* = internal light path in cm and *c* = concentration of K_2_Cr_2_O_7_ solution in g kg^−1^. The absorptivities computed for the five concentrations of K_2_Cr_2_O_7_ solutions used are summarized in table II. The values tabulated were determined from least-squares plots of the experimental values and were subsequently normalized to the concentrations shown. The uncertainties given include all known sources of possible systematic error and the 95 percent (2*σ*) confidence interval for the mean. The random component of these uncertainties, based on standard deviations computed from residuals resulting from fitting the data to the various concentration levels for each wavelength, is 0.07 percent at the 2*σ* level.

It is observed that all absorptivities in table II increase with increasing K_2_Cr_2_O_7_ concentration. These deviations from Beer’s law are produced by the fact that, in weakly acidic media, chromium (VI) ions exist as two distinct absorbing species—HCrO_4_^–^ and its dimerization product, Cr_2_O_7_^=^. The equilibrium between these two species may be represented as

$$2{\text{HCrO}}_{4}{\;}^{-}\overset{K_{D}}{=}{\text{Cr}}_{2}O_{7}{\;}^{=}+H_{2}O$$ (1)

and the corresponding dimerization constant, *K_D_*, is given by

$$K_{D}=\frac{\left[{\text{Cr}}_{2}O_{7}{\;}^{=}\right]}{{\left[{\text{HCrO}}_{4}{\;}^{-}\right]}^{2}}$$ (2)

Although eq. (2) predicts that the formation of Cr_2_O_7_^=^ is strictly a quadratic function of K_2_Cr_2_O_7_ concentration, the value of *K_D_* = 32.9 (mol kg^−1^)^−1^ obtained previously [3] is of such magnitude that the percentage of total chromium present as the Cr_2_O_7_^=^ ion is very nearly a linear function of the K_2_Cr_2_O_7_ concentration for the range of solutions studied. Calculated values of HCrO_4_^–^/Cr_2_O_7_^=^ speciation in this system are given in table III.

**Figure 3:**
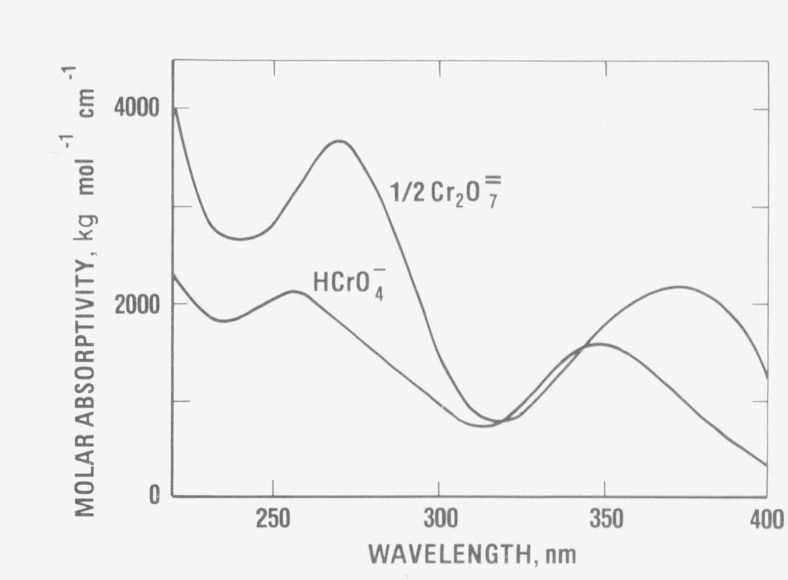
Absorbance spectra of the *HCrO*_4_^–^ ion and its dimerization product, *Cr*_2_*O*_7_^=^

The direction and magnitude of the expected deviations from Beer’s law for the acidic potassium dichromate system can be ascertained when the speciation data in table III are combined with the spectral characteristics of the two chromium (VI) species. Figure 3 shows the relation of the absorbance spectra of the HCrO_4_^–^ and Cr_2_O_7_^=^ ions. At wavelengths of 235, 257, 313 and 350 nm, the Cr_2_O_7_^=^ ion is shown always to have a larger absorptivity than the HCrO_4_^–^ ion. Hence, the measured absorptivities at these wavelengths should increase with increasing K_2_Cr_2_O_7_ concentration.

From the same argument, the data in figure 3 also suggest that there are two wavelengths near 320 and 345 nm where Beer’s law is obeyed. In an attempt to determine these isosbestic points experimentally, absorbance measurements were also made on the same solutions used above at 322, 323, 345 and 346 nm. The corresponding absorptivity values are given in table IV. Although small systematic deviations from Beer’s law are still present, the absorptivities calculated for 345 nm are sufficiently constant that the acidic K_2_Cr_2_O_7_ system can be used at this wavelength, over the concentration range shown, to check the absorbance linearity of narrow bandpass spectrophotometers to one part in a thousand.

The variation of absorptivity of the acid K_2_Cr_2_O_7_ system with temperature is the smallest that we have observed for any liquid filter that we have studied to date. For the four wavelengths recommended for checking the accuracy of the absorbance scale—235, 257, 313, and 350 nm—the absorptivities decrease with increasing temperature. Over the range 20–30 °C, the corrections are, respectively, −0.05, −0.05, −0.02, and −0.05 percent per degree Celsius. The −0.02 percent correction found at 313 nm corresponds closely to the correction predicted from the thermal expansion of the solvent. Until further evaluation can be made of the temperature dependence of 345 nm wavelength recommended for checking absorbance linearity, measurements should be restricted to 23.5 ± 1 °C.

Several considerations were involved in the selection of perchloric acid for acidifying the K_2_Cr_2_O_7_ solutions. First, perchloric acid was preferred over the sulfuric acid which has been used traditionally because perchlorate ion, unlike sulfate, has no tendency to form mixed complexes with chromium (VI) species. Secondly, the choice of 0.001 *M* acid rather than 0.01 *M* was based on two factors: (1) the 0.001 *M* acid represented a practical compromise for minimizing conversion of HCrO_4_^–^ to either H_2_CrO_4_ or CrO_4_^=^ and (2) the lower acid concentration substantially lowered the oxidation potential (~13 percent) of the chromium (VI) ions and hence improved the solution stability of the proposed K_2_Cr_2_O_7_ standards.

**Figure 4:**
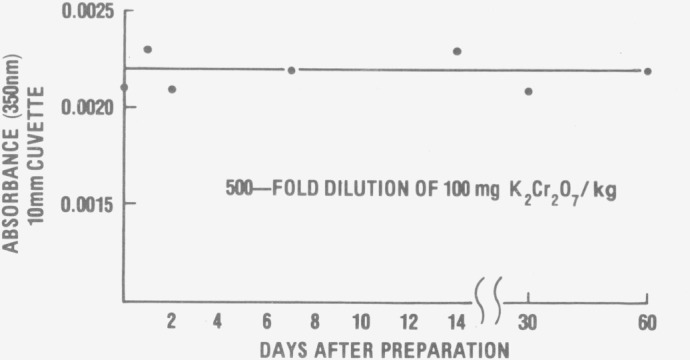
Test of the distilled water for reducing impurities.

A final point that needs to be emphasized concerns the quality of the distilled water used for preparing the standard solutions. Acidic potassium dichromate is a potential oxidant and consequently the distilled water used must be shown to be free of reducing impurities in order that the absorptivity data given in this paper be valid. A simple but yet definitive test is outlined in figure 4. In this study a 500-fold dilution of a 100 mg kg^−1^ standard solution of K_2_Cr_2_O_7_ was made with the distilled water in question and its absorbance measured at 350 nm. Not only did the measured absorbance agree with the predicted value, thus indicating no reduction of chromium (VI), but also subsequent measurements showed that this K_2_Cr_2_O_7_ solution standard should be stable for at least two months provided it is adequately protected against evaporation. In instances where this test shows the distilled water to be suspect, it is recommended that the water be redistilled from alkaline potassium permanganate in order to preoxidize the impurities.

**Figure 5:**
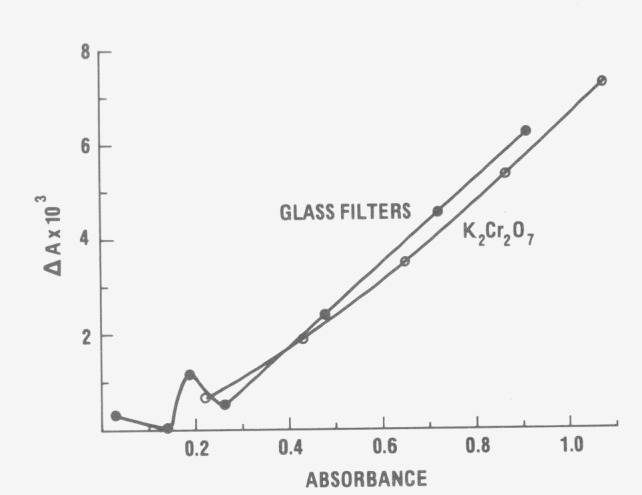
Correction Δ*A* for the absorbance scale of a precision commercial spectrophotometer.

Finally, the general use of solid and liquid transfer standards for calibrating the absorbance scale of a precision commercial spectrophotometer is illustrated in figure 5. The measurements on the glass filters were performed at 440 nm while the K_2_Cr_2_O_7_ measurements were made at 350 nm. At both wavelengths the absorbances measured on the precision spectrophotometer are high and should be corrected by subtracting the appropriate Δ*A* values.

## IV. Conclusion

Following widespread use in the collaborative testing of spectrophotometers for more than 25 years, the absorptivities of the acidic potassium dichromate system have now been determined with a known accuracy from measurements performed directly on the NBS Institute for Materials Research high- accuracy spectrophotometer. Later this year, crystalline K_2_Cr_2_O_7_, together with detailed instructions on preparing solutions from it, will be available from NBS through its Office of Standard Reference Materials as an SRM. In conjunction with the calibrated quartz cuvettes previously issued (SRM 932), it is believed that this material will provide a valid check of the accuracy of the absorbance scale of narrow bandpass spectrophotometers in the ultraviolet from 235–350 nm.

## Figures and Tables

**Table I:** Reproducibility of absorbance measurements at 235 nm for a cycle of solvent and sample runs

Cuvette No.	Solvent	Sample	Sample	Solvent
	After 5 rinses	After 5 rinses	After 7 rinses	After 5 rinses
				
14	0.05198	0.30128	0.30141	0.05213
16	.05099	.55052	.55057	.05093
18	.05310	.80066	.80074	.05321
25	.05002	1.04863	1.04855	.05022
26	.05064	1.30351	1.30333	.05065
Ref. filter^a^	.55913	.55925	.55936	.55922

a Inconel-on-quartz.

**Table II:** Absorptivities of K_2_C_2_O_7_ in 0.001 M perchloric acid at 23.5 °C Absorptivity, kg g^−1^cm^−1^

K_2_Cr_2_O_7_ Conc., g kg^−1^	235 (1.2)^b^ nm	257 (0.8) nm	313 (0.8) nm	350 (0.8) nm	Uncertainty^c^
					
a0.020	12.243	14.248	4.797	10.661	0.034
.040	12.291	14.308	4.804	10.674	d.022
.060	12.340	14.369	4.811	10.687	d.020
.080	12.388	14.430	4.818	10.701	d.020
.100	12.436	14.491	4.825	10.714	d.019

a Nominal concentration; all weights corrected to vacuum.

b Wavelength and, (), spectral bandwidth.

c Includes estimated systematic errors and the 95 percent confidence interval for the mean.

d For wavelength of 313 nm, the uncertainty is reduced to half of these values for K_2_Cr_2_O_7_ concentrations of 0.040, 0.060, 0.080 and 0.100 g kg^−1^.

**Table III:** HCrO_4_^–^/Cr_2_O_7_^=^ speciation in 0.001 M perchloric acid solutions of K_2_Cr_2_O_7_.

K_2_Cr_2_O_7_ Conc., g kg^−1^	Percent Cr as HCrO_4_^–^	Percent Cr as Cr_2_O_7_^=^
		
0.020	99.10	0.90
.040	98.22	1.78
.060	97.38	2.62
.080	96.56	3.44
.100	95.77	4.23

**Table IV:** Absorptivities of K_2_Cr_2_O_7_ in 0.001 M perchloric acid near two predicted isosbestic wavelengths; temperature 23.5 °C Absorptivity, kg g^−1^ cm^−1^

K_2_Cr_2_O_7_ Conc., g kg^−1^	322 (0.8)^b^ nm	323 (0.8) nm	345 (0.8) nm	346 (0.8) nm
				
a0.020	5.845	6.065	10.593	10.615
.040	5.842	6.062	10.595	10.621
.060	5.838	6.059	10.598	10.627
.080	5.835	6.056	10.600	10.632
.100	5.831	6.053	10.602	10.638

a Nominal concentration; all weights corrected to vacuum.

b Wavelength and, (), spectral bandwidth.

## Footnotes

1 Figures in brackets indicate the literature references at the end of this paper.

2 In order to describe materials and experimental procedures adequately, it is occasionally necessary to identify the sources of commercial products by the manufacturer’s name. In no instance does such identification imply endorsement by the National Bureau of Standards, nor does it imply that the particular product is necessarily the best available for that purpose.
